# Development of Simple Designs of Multitip Probe Diagnostic Systems for RF Plasma Characterization

**DOI:** 10.1155/2014/279868

**Published:** 2014-02-05

**Authors:** M. Y. Naz, S. Shukrullah, A. Ghaffar, N. U. Rehman

**Affiliations:** ^1^Department of Fundamental and Applied Sciences, Universiti Teknologi PETRONAS, 31750 Tronoh, Perak, Malaysia; ^2^Department of Physics, University of Agriculture, Faisalabad 38040, Pakistan; ^3^Department of Electrical Engineering, King Saud University, Riyadh 11451, Saudi Arabia; ^4^Department of Physics, COMSATS Institute of Information Technology, Islamabad, Pakistan

## Abstract

Multitip probes are very useful diagnostics for analyzing and controlling the physical phenomena occurring in low temperature discharge plasmas. However, DC biased probes often fail to perform well in processing plasmas. The objective of the work was to deduce simple designs of DC biased multitip probes for parametric study of radio frequency plasmas. For this purpose, symmetric double probe, asymmetric double probe, and symmetric triple probe diagnostic systems and their driving circuits were designed and tested in an inductively coupled plasma (ICP) generated by a 13.56 MHz radio frequency (RF) source. Using *I*-*V* characteristics of these probes, electron temperature, electron number density, and ion saturation current was measured as a function of input power and filling gas pressure. An increasing trend was noticed in electron temperature and electron number density for increasing input RF power whilst a decreasing trend was evident in these parameters when measured against filling gas pressure. In addition, the electron energy probability function (EEPF) was also studied by using an asymmetric double probe. These studies confirmed the non-Maxwellian nature of the EEPF and the presence of two groups of the energetic electrons at low filling gas pressures.

## 1. Introduction

The low pressure discharge plasma can be excited and sustained by different plasma sources [1–3] and ICP source is one of them [4, 5]. In ICPs, the plasma chemistry is mainly controlled by the gas temperature and electron energies [1, 6]. It reveals that the role of electron temperature and number density is very important in order to understand the phenomena of electron impact ionization and excitation processes [7, 8]. The phenomena of low pressure plasma discharges become very complex during the conversion of electron energy into some other forms such as chemical energy, sound energy, light energy, and heat. In ICPs, the energetic electrons have frequent collisions with each other and with other plasma species and remain in an equilibrium state with a definite electron energy distribution function (EEDF) and electron energy probability function (EEPF) [1]. For the Maxwellian energy distribution, the semilogarithmic plot of EEPF is a straight line which reveals that it is more important and simple to analyze in comparison with corresponding EEDF. In recent years, a lot of work has been done in order to fully characterize the ICPs [7] and many efforts are under way for the measurement of plasma parameters like electron number density (*n*_*e*_), ion number density (*n*_*i*_), electron temperature (*kT*_*e*_), plasma potential (*V*_*s*_), electron saturation current (*I*_eo_), ion saturation current (*I*_io_), electron energy distribution function (EEDF), and so forth in a very precise way [8].

Different tools and techniques [7, 9] can be employed to characterize the discharge plasma but the electrostatic probes are considered to be the most powerful and experimentally simple technique for plasma characterization over a wide range of plasma densities because they did not require the assumption that the plasma should be in local thermodynamic equilibrium. Irvin Langmuir was the first who measured the volt-ampere characteristics by inserting a single conducting wire into plasma and then using it; he determined the electron temperature and plasma density [10]. This simple device nowadays commonly used in plasma measurements is called the single Langmuir probe. The electrostatic probes are normally composed of DC measurement circuits and metallic tips and therefore are more convenient method of plasma characterization as compared to other methods such as laser induced fluorescence, optical emission spectroscopy, and microwave interferometry. The low temperature plasma chambers generally have no well-defined plasma grounds. In this plasma, the multitip probes are more frequently used than the single probe. The single probe has few main drawbacks which are difficult to overcome particularly when the reference electrode is absent or when the plasma potential is not well defined. Moreover, unless the probe area is sufficiently small, it may draw large electronic current by disturbing the discharge conditions, when operated close to the space potential. It shows that the single probe method may not be suitable for decaying plasma accompanied by the perturbations. In order to meet these difficulties introduced by single probes, double and triple probes of different geometries are being developed and tested in low pressure discharge plasma. Multitip probes are found to have negligible influence on tested plasma and yield accurate data while investigating different plasma parameters [11].

Therefore, in this detailed note, symmetric and asymmetric double probes and a symmetric triple probe were manufactured using nickel-chrome wire tips and ertalon casing. These probes were used to characterize the inductively coupled nitrogen plasma generated by using a 13.56 MHz RF source. The nitrogen discharge plasma parameters such as *kT*_*e*_, *n*_*e*_, *I*_io_, and EEPF were determined as the function of changing input power and filling gas pressure. Then a comparison was carried out between the results obtained by all three probes which showed a good agreement in all measurements.

## 2. Multitip Probe Theory

### 2.1. Symmetric Double Probe (SDP)

**Figure 1:**
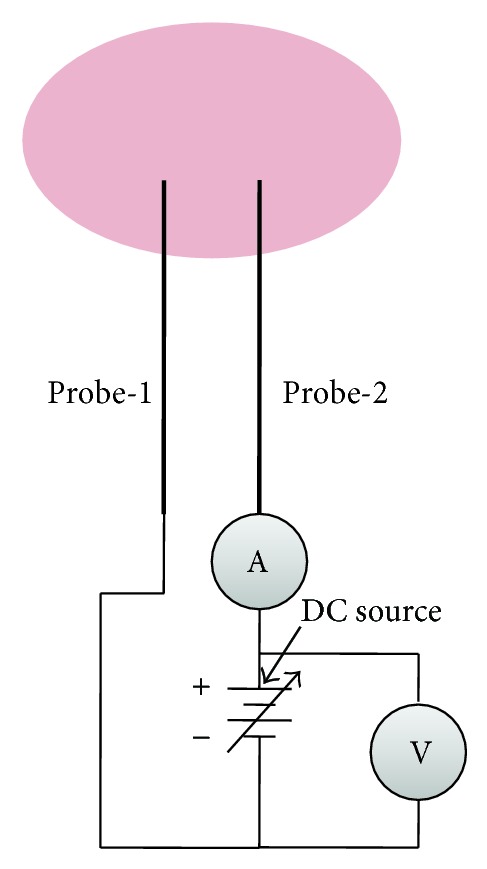
Schematic of double Langmuir probe.

A SDP is composed of a pair of single probes of the same shape, geometry, and size. A direct current biasing source is connected across these small probes as shown in Figure 1. If the electrons in discharge plasma obey the Maxwellian energy distribution, then the plasma parameters can be evaluated from the *I*-*V* characteristic curve of SDP by using the following equation [1, 5, 6]:

(1) $$\begin{array}{c} {\left(\frac{dI}{dV_{d}}\right)}_{V_{d=0}}=\frac{eI_{\text{io}}}{2kT_{e}}. \end{array}$$

The above equation (1) shows that the slope of the *I*-*V* characteristic curve at zero relative DC biasing voltage provides *kT*_*e*_ and *I*_io_, whereas *n*_*e*_ can be determined by using these *I*_io_ and *kT*_*e*_ values in the following equation:

(2) $$\begin{array}{c} I_{\text{io}}=0.61n_{e}eA_{p}\sqrt{\frac{kT_{e}}{m_{i}}}. \end{array}$$

While determining the *kT*_*e*_ from the *I*-*V* characteristic curve of SDP, it was assumed that the high energy electrons obey the Maxwellian distribution in order to overcome the retarding field of probes. But in the real case, it was observed that the most of the discharge plasma obeys the non-Maxwellian energy distribution and SDP gives more erroneous data during the characterization of such plasma [4].

### 2.2. Asymmetric Double Probe (ADP)

The electrons collected by the SDP come from the high energy tail of electron energy distribution probability (EEDP). Therefore, the electrons in these types of discharge plasma have Maxwellian distribution and *kT*_*e*_ determined by SDP shows close agreement with the measurements made with single probe which has ability to scan the full energy range. Hence, it is possible to determine the electron energy distribution function (EEDF) and EEPF only with the single Langmuir probe method but not with SDP. This small discrepancy can be overcome by changing probe symmetry. For the collection of majority of the electrons by the probe from the whole energy distribution, the area of the one probe is increased several hundred of times more than the other one. By doing so, the EEDF can be determined by taking the second derivative of the electron current (*I*_*e*_) with respect to repelling potential given as [4]

(3) $$\begin{array}{c} F_{e}\left(\varepsilon \right)=\frac{4}{A_{P}e^{2}}\sqrt{\frac{m_{e}V}{2e}}\frac{d^{2}I_{e}}{dV^{2}}. \end{array}$$

In this equation, *ε* = *eV* = *e*(*V*_*p*_ − *V*_*s*_) is the electron energy, *I*_*e*_ = *I*_*p*_ − *I*_io_ is the electron current, *m*_*e*_ is the electron mass, *A*_*p*_ is the probe area, *V*_*s*_ is the space potential, and *I*_*p*_ is the probe current. If the plasma electrons have the Maxwellian energy distribution, the log plot of EEPF will be straight. It means that EEPF is easier to analyze than the corresponding EEDF. Hence, using (3), we can determine the EEPF as

(4) $$\begin{array}{c} F_{p}\left(\varepsilon \right)=\varepsilon^{-1/2}F_{e}\left(\varepsilon \right). \end{array}$$

The average electron energy or electron temperature also called the effective electron temperature can be determined by

(5) $$\begin{array}{c} \langle \varepsilon \rangle =\frac{3}{2}kT_{e}, \end{array}$$

(6) $$\begin{array}{c} kT_{e}=\frac{2}{3}\langle \varepsilon \rangle . \end{array}$$

However, ADP cannot be used to determine *n*_*e*_ in the plasma having non-Maxwellian EEDP.

### 2.3. Symmetric Triple Probe (STP)

**Figure 2:**
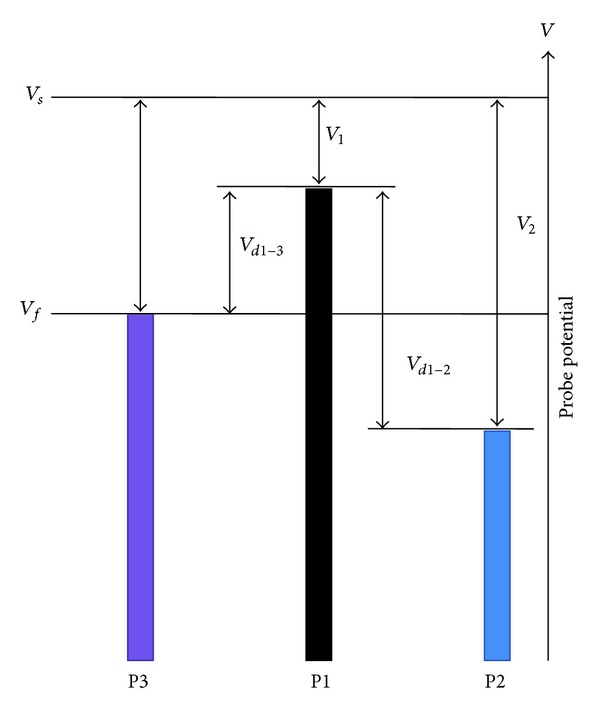
Potential of each probe with respect to *V*_*s*_ in triple probe arrangement.

Although single and double probes are very good tools for the plasma characterization, they require the voltage sweep to obtain corresponding *I*-*V* characteristics, which limits the time resolution of the measurements. This factor makes double probes difficult to characterize the time varying plasma. The issue can be resolved by using a STP [11] composed of three plasma exposed metallic tips as shown in Figure 2, where *V*_*d*1−2_ is the biasing voltage applied across probe-1 and probe-2 and *I*_1_ is the corresponding probe current, while *V*_*d*1−3_ is the potential difference across probe-1 and the floating probe-3. For precise measurement of *kT*_*e*_, biasing voltage should obey the condition *V*_*d*1−2_ ≥ 2*kT*_*e*_. In these studies, a uniform space potential *V*_*s*_ was assumed in the sampling region of the characterized plasma. The potential difference of all three probes with respect to *V*_*s*_ is also explained in Figure 2.

The current through each probe can be written as

(7) $$\begin{array}{c} I_{p1}=I_{1}=I_{\text{io}}+I_{\text{eo}}\exp\left(-\frac{eV_{1}}{kT_{e}}\right), \end{array}$$

(8) $$\begin{array}{c} I_{p2}=I_{2}=I_{\text{io}}+I_{\text{eo}}\exp\left(-\frac{eV_{2}}{kT_{e}}\right), \end{array}$$

(9) $$\begin{array}{c} I_{p3}=0=I_{\text{io}}+I_{\text{eo}}\exp\left(-\frac{eV_{3}}{kT_{e}}\right). \end{array}$$

Dividing (7) by (8) and simplifying, we get

(10) $$\begin{array}{c} 2\exp\left(-\frac{eV_{d1-3}}{kT_{e}}\right)=1+\exp\left(-\frac{eV_{d1-2}}{kT_{e}}\right). \end{array}$$

In the limit *eV*_*d*2_ > >2*kT*_*e*_, (10) will become as

(11) $$\begin{array}{c} kT_{e}=\frac{eV_{d1-3}}{\ln2}=\frac{e\left(V_{1}-V_{f}\right)}{\ln2}. \end{array}$$

*kT*_*e*_ can be calculated by using (11). Obviously, it is not reasonable to ignore the term exp⁡(−*V*_*d*1−2_/*kT*_*e*_) in (10) in the limit of *eV*_*d*2_ > >2*kT*_*e*_, without considering *V*_*d*1−2_/*V*_*d*1−3_ ratio.

Now *n*_*e*_ can be obtained from (6) [11]:

(12) $$\begin{array}{c} n_{e}=\frac{1}{0.61Ae\sqrt{kT_{e}/m_{i}}}I_{\text{io}}. \end{array}$$

In case of STP, *I*_io_ can be obtained after subtracting (9) from (7) and simplifying them:

(13) $$\begin{array}{c} I_{\text{eo}}=I_{1}\frac{\exp\left(-eV_{d1-3}/kT_{e}\right)}{\left(1-\exp\left(-eV_{d1-3}/kT_{e}\right)\right)}. \end{array}$$

Now, (12) can be modified as

(14) ne=−I10.61AekTe/miexp⁡(−eVd1−3/kTe)(1−exp⁡(−eVd1−3/kTe)) ∵−Iio=Ieo.

This equation (14) is used to measure *n*_*e*_ in discharge plasma by suing STP method.

## 3. Materials and Methods

**Figure 3:**
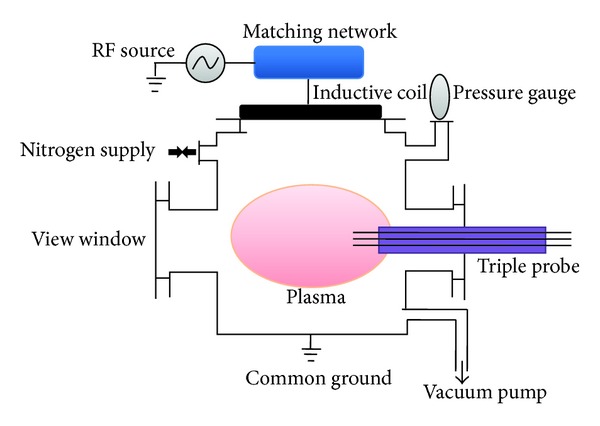
Schematic of the experimental setup.

Schematic of the experiment setup used for the generation and characterization of ICP with nitrogen as a base gas is shown in Figure 3. The nitrogen discharge plasma was generated and sustained by 13.56 MHz RF [12] source of 50 *Ω* resistance, connected across a spiral planar coil shaped electrode. This copper electrode of 13 turns and 30 cm diameter was placed at the top opening of a cylindrical plasma chamber. The stainless steel plasma chamber was 31 cm in diameter and 24 cm in height with four main vacuum tight multirole ports of 9.8 cm diameter each. The plasma chamber was insulated from the spiral planar coil electrode by placing a 1.2 cm thick quartz plate between them. The quartz plate was not only serving as an insulator between the electrode and plasma chamber but also helped in maintaining vacuum inside the chamber. In order to keep the power reflection below 2% and to maximize the power transfer from the RF generator to discharge gas, an automatic impedance matching network composed of a tuning unit and a control unit was employed between an RF generator and a copper electrode. For flow mode operation and low pressure plasma generation, a rotary vane pump capable of lowering the pressure up to 10^−3^ bar was coupled with plasma chamber. The gas flow rate from the main cylinder to plasma chamber was controlled by a mass flow meter and chamber pressure was monitored by using a Pirani gauge. In this experiment, the nitrogen gas flow rate was kept constant at 50 sccm throughout.

### 3.1. Development of Multitip Probes

**Figure 4:**
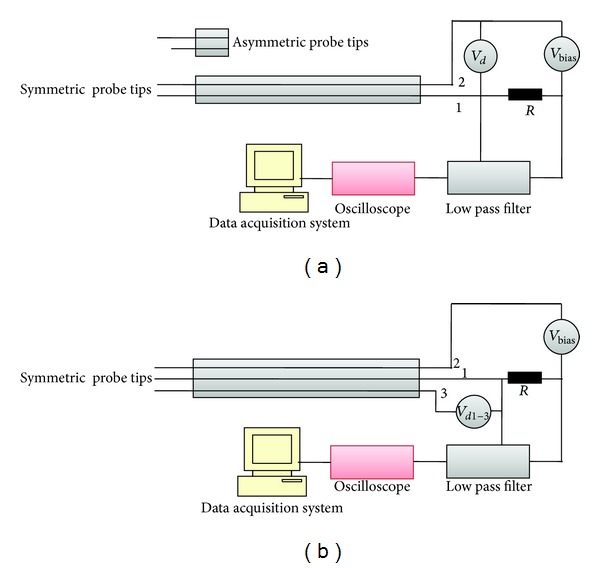
(a) Symmetric and asymmetric double probes design. (b) Symmetric triple probe design.

The careful design of the probe and associated electric circuit is very important for successful operation of multitip probes and characterization of discharge plasma. To verify the compatibility of the probe dimensions chosen for a particular experiment, the probe theory presented in this note must be valid during the determination of expected parameters. Keeping this in mind, the SDP, ADP, and STP systems were developed in laboratory for characterization of nitrogen discharge plasma. The schematics of the probe designs are given in Figures 4(a) and 4(b). SDP was composed of two plasma exposed nickel-chrome tips of the same length, radius, and surface area. Each exposed tip was 11 mm in length, 0.22 mm diameter, and 7.59 mm^2^ surface area. ADP was composed of two plasma exposed tips of the same material but not of the same dimension as in the case of SDP. One of the two exposed tips had the surface area of 2.76 mm^2^ which was 94.66 times smaller than the 2nd tip (261.25 mm^2^). STP was also made of three exposed tips of the same length and diameter. These tips were 0.22 mm in diameter and 7.59 mm^2^ area. The probe casing was made of 17.5 cm long ertalon tube with an inner diameter of 6 mm. For all probes, this vacuum tight tubing was not only insulating the wires from the plasma chamber but also keeping the tips at a fixed separation. Outside of the plasma chamber, these probes were connected to BNC coaxial cables running along the probe arm and down to an insulated BNC feed through. These coaxial cables pass the electrical signal from probe tip to the digital oscilloscope through auxiliary circuit arrangements. A DC biasing source (+120 V to −120 V) was also connected across the probes for manual scanning from +26 V to −26 V and corresponding current through the probe circuit was measured [13, 14]. For the elimination of RF noise picked up by data cables, the RC low pass filters were used at the inputs of the oscilloscope. Outside the plasma chamber, the probe shielding was connected to the chamber wall and a single common ground was provided to the whole setup.

## 4. Experimental Results

**Figure 5:**
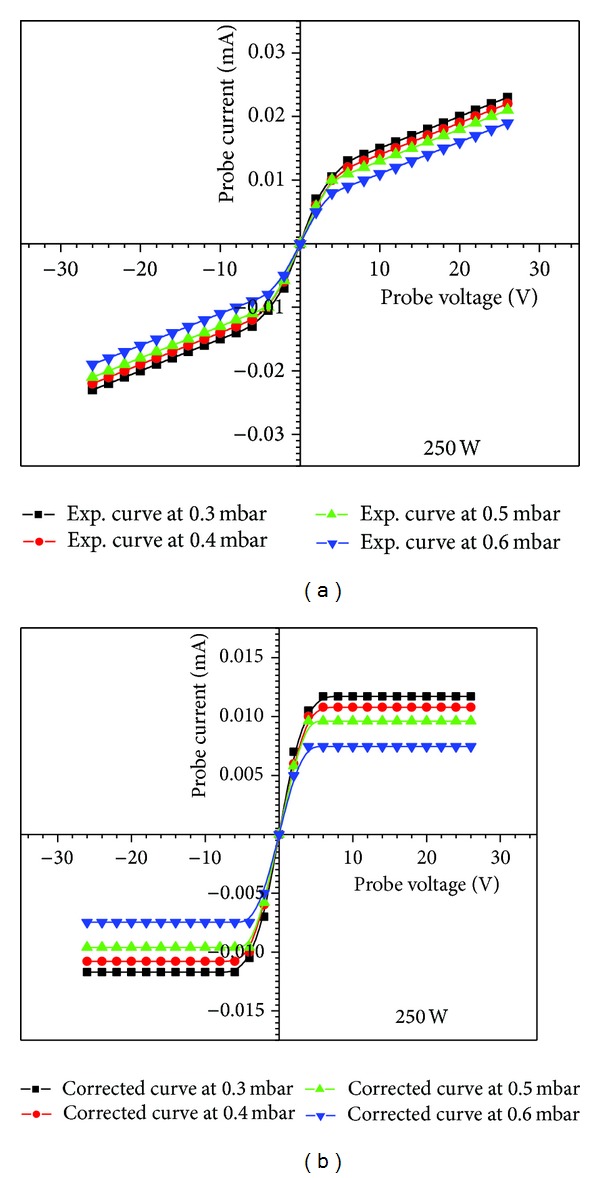
(a) Symmetric double probe *I*-*V* characteristics for different filling gas pressures. (b) The corrected *I*-*V* characteristics for different filling gas pressures.

The *I*-*V* characteristics curve of SDP at 250 W input RF power and 0.3, 0.4, 0.5, and 0.6 mbar filling pressures are shown in Figure 5(a). For SDP, *kT*_*e*_ and *I*_io_ were obtained from the slope of the *I*-*V* characteristics at zero relative bias voltage (*V*_*d*_) and by using (1), whereas *n*_*e*_ was obtained by using *kT*_*e*_ values in (2). As the current from plasma to the probe may be due to diffusion, convection, high energy electrons, ions, and electric field of the probe sheath [15], therefore slope of the *I*-*V* characteristics should satisfy (1) for accurate measurement of *kT*_*e*_ and *n*_*e*_. Therefore, few corrections were made by fitting a straight line to ion saturation current regimes in *I*-*V* characteristics as shown in Figure 5(b), where its intersection with ordinate gives *I*_io_ and resultant slope satisfies (1).

**Figure 6:**
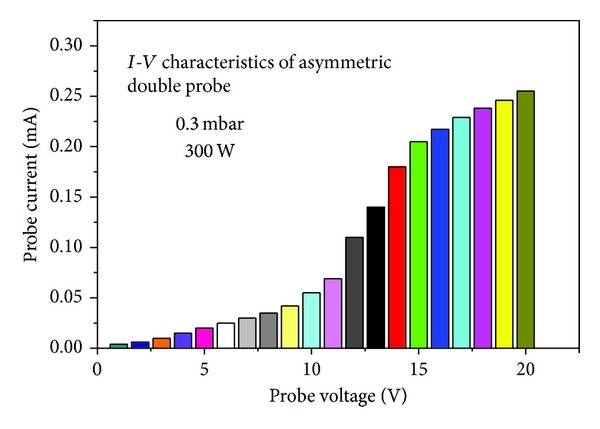
Asymmetric double probe *I*-*V* characteristics at 300 W RF power.

The typical *I*-*V* characteristics curve of ADP obtained at 300 W input power and 0.3 mbar chamber pressure is shown in Figure 6. As ADP tips receive the different electrons fluxes, therefore the characteristics curve does not pass through origin of the coordinate system. In such type of *I*-*V* characteristic curves, *I*_io_ may be determined in a similar way to single probes. The turning point of the curve gives space potential which is used to determine the electron current (*I*_*e*_ = *I*_*p*_ − *I*_io_) and repelling potential (*V* = *V*_*s*_ − *V*_*p*_). Using these parameters in (4), (5), and (6), we can evaluate EEPF, 〈*ε*〉, and *kT*_*e*_.

While using STP, *kT*_*e*_ was obtained from (11) under same plasma operating conditions as in the case of SDP and ADP. These *kT*_*e*_ values were used in (14) to obtain *n*_*e*_ in the limit of *eV*_*d*1−2_ > >2*kT*_*e*_. In (11), *V*_*d*1−3_ is the voltage difference between the probe-1 and floating probe-3. This voltage difference and the current through the probe-1 are the functions of biasing voltage difference *V*_*d*1−2_ which is applied between the probe-1 and probe-2. By the limit *eV*_*d*1−2_ > >2*kT*_*e*_, we mean that the probe currents in this experiment were saturated at maximum voltage of +4.1 V and −4.1 V. So, the plasma potential should be less than 4.1 V because electrons in the plasma were moved from the plasma to the probes by the electric force of the applied saturation voltages.

## 5. Discussions

**Figure 7:**
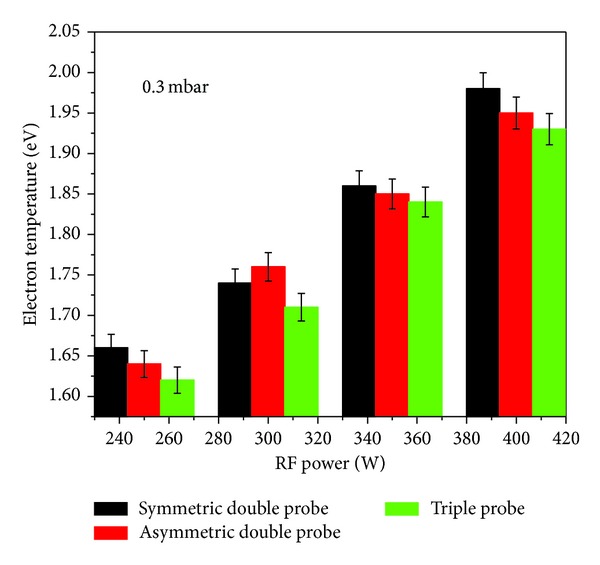
The electron temperature as a function of input RF power.

**Figure 8:**
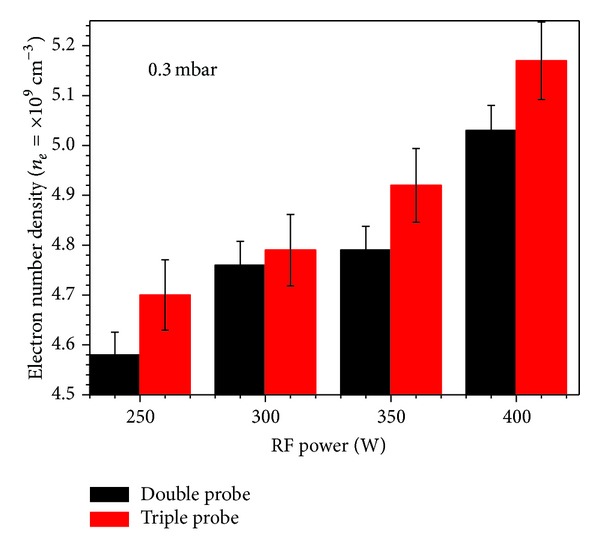
The electron number density as a function of input RF power.

The graphical representation of the results for *kT*_*e*_ and *n*_*e*_ as a function of input RF power is given in Figures 7 and 8, respectively. A monotonic increase in *kT*_*e*_ and *n*_*e*_ was noticed with rise of input power from 250 to 400 W at a fixed pressure of 0.3 mbar [16]. This increasing trend in *kT*_*e*_ may be due to an increase in kinetic energy of the electrons under the influence of increasing incident RF power. As for us *n*_*e*_ is concerned, although it reciprocally depends on *kT*_*e*_ as mentioned in the probe theory, in our case, such increase might be due to an increase in ionization events in the discharge plasma at higher powers, which results in an increase of the ion saturation current having the direct relation with number density [17]. As the input power increases, the energy available for discharge plasma also increases and consequently the ionization events, which results in large ionization probabilities especially at low filling gas pressures.

**Figure 9:**
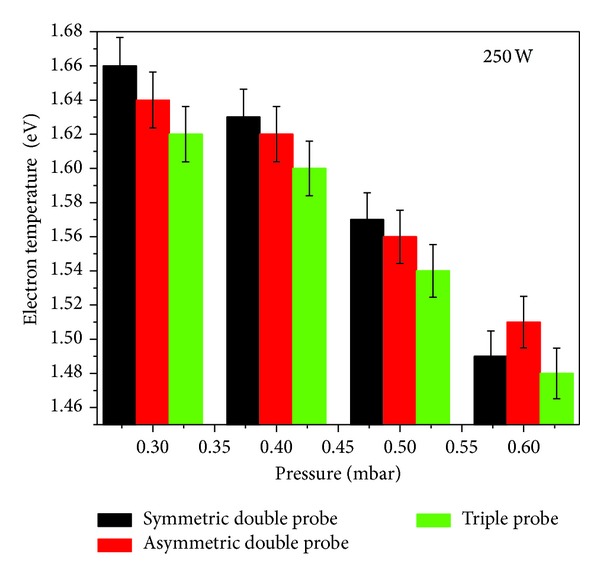
The electron temperature as a function of filling gas pressure.

**Figure 10:**
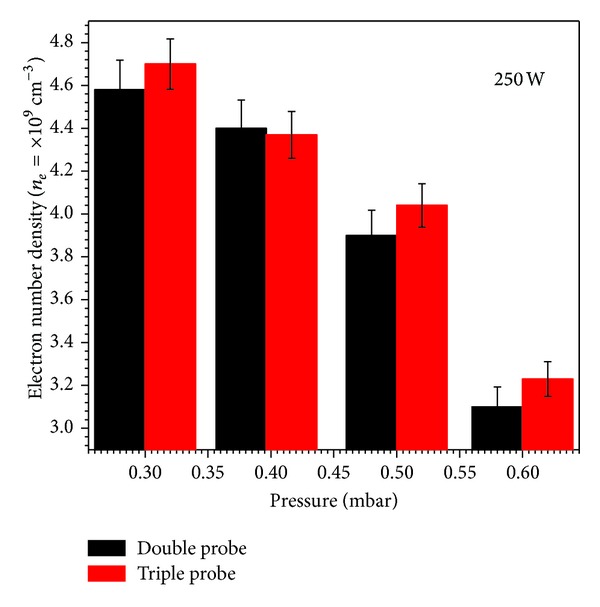
The electron number density as a function of filling gas pressure.

The variational trend in *kT*_*e*_ and *n*_*e*_ with filling gas pressure at constant input power of 250 W is shown in Figures 9 and 10. It was noticed that *kT*_*e*_ decreases with increase in filling gas pressure. This decreasing trend in the electron temperature might be due to the following reasons. The increase in filling gas pressure inside the chamber brings about a high cooling frequency in the plasma chamber which causes a decrease in electron energy and consequently *kT*_*e*_ [17]. The filling gas pressure and input power dependence of average electron energy 〈*ε*〉 and hence *kT*_*e*_ can also be explained by the steady state particle balance equation due to a linear relationship between the average electron energy and the effective electron temperature. When the filling pressure inside the chamber increases, the frequency of electron collisions with the plasma species also increases and the mean free path between successive collisions decreases. It shows that despite gaining the energy, the electrons lose their energy in the discharge. It means that as the chamber pressure increases, more and more energy is transferred from the electrons to the plasma species. In such cases, a balance between the total ionization events and the total particle losses to the chamber walls [18] is difficult to exist which results in decrease of *kT*_*e*_ and an increase in the neutral gas temperature at higher filling gas pressures [19]. A similar decreasing trend was noticed in *n*_*e*_ determined with SDP and STP as a function of filling gas pressure at constant input power of 250 W. For the SDP method, these calculations were made in the low current portion of the *I*-*V* characteristics to minimize the effect of RF fluctuations. This decreasing trend in *n*_*e*_ may be due to the following reasons. An increase in filling gas pressure inside the plasma chamber causes a cooling effect on the discharge plasma which results in a decrease of the ion saturation current and consequently *n*_*e*_ [17]. At higher pressures and relatively low input powers, the elastic collisions of the electrons with the plasma species can also play a significant role in the reduction of ionization events. Similarly, at higher pressures, the high energy tail of EEDF depletes to lower energies; therefore the availability of the highly energetic electrons for electron impact ionization processes decreases and consequently *n*_*e*_. This depletion in the tail of electron energy distribution function might be due to the rapid diffusion and recombination of highly energetic electrons at the chamber walls [19].

Although the graphical representation of the results obtained with SDP, ADP, and STP methods shows very close agreement, the *kT*_*e*_ obtained with ADP was relatively higher followed by SDP and STP. It might be due to the following reasons. The double probes operate close to the floating potential and measure only the high energy electrons in the tail of the EEDF. These highly energetic electrons are least influenced by the probe electric field at relatively small DC biasing voltage and cause an inaccuracy in the determination of the slope of the *I*-*V* characteristics in the retardation region. This inaccuracy in slope determination normally can range from 10% to 25%. In order to overcome this problem, a highly sensitive driving circuit is required by the double probes especially in case of SDP for voltage sweep and to obtain the *I*-*V* characteristics. It makes the probe system not only complicated and susceptible to the disturbances in the plasma but also causes the grounding problem. Moreover, any possible asymmetry in probe tips may change the probe dimensions, causing a shift in *I*-*V* characteristics and consequently in plasma parameters.

**Figure 11:**
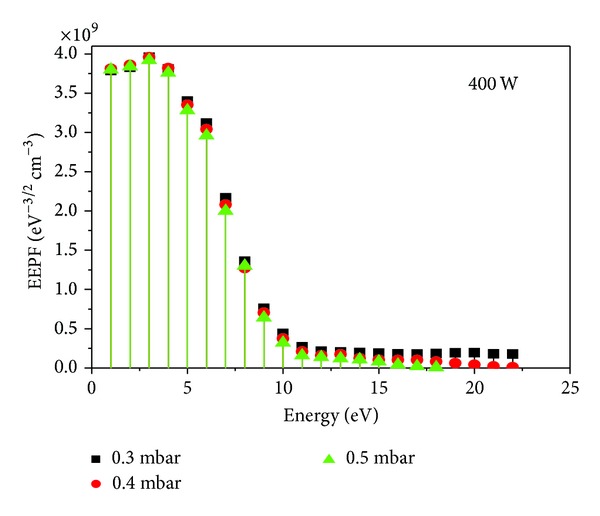
EEPF determined with an asymmetric double probe at 450 W input RF power and different filling gas pressures.

In addition to *kT*_*e*_ and *n*_*e*_, EEPF was also investigated in these studies. EEPF is easier to analyze than corresponding EEDF and was characterized with ADP at 0.3, 0.4, and 0.5 mbar filling gas pressure and 400 W input power as shown in Figure 11. It is clear from the plot that the high energy tail of EEPF increases with the decrease of filling gas pressure, showing its non-Maxwellian behavior. This increment in the tail confirms the presence of a large number of highly energetic electrons in the discharge plasma [18] along with the least energetic electrons. But at higher filling pressures, the high energy tail of the EEPF depletes to the lower energies indicating the Maxwellian approach of EEPF. This depletion in the high energy tail may be due to the rapid diffusion and recombinations of highly energetic electrons at the chamber walls. The electrons have random motion and undergo inelastic collisions with plasma species. During these collisions, they exchange their energies with other particles, resulting in reduction of fast electrons near the probes and consequently the high energy tail [1, 4].

## 6. Conclusions

In this detailed note, the multitip Langmuir probes along with their necessary driving circuits were developed successfully for characterization of low pressure inductively coupled nitrogen plasma. The results of the probe measurements were compared to check the validity of these methods. First, electron temperature was measured by using a symmetric double probe, asymmetric double probe, and triple probe and electron number density by using a symmetric double probe and triple probe at different filling gas pressures and input RF powers. From the results, it was depicted that at a fixed pressure of 0.3 mbar, both the electron temperature and the electron number density increased with the increase of input RF power. The relative inaccuracy in measurement of electron temperature and electron number density with all three probes ranged from 3% to 15% and 3% to 16%, respectively. Similarly, the variational trend of electron temperature and electron number density was also studied as a function of filling gas pressure (0.3 to 0.6 mbar) at a fixed input RF power of 250 W. It was found that not only electron temperature as expected but electron number density also showed the same decreasing trend. The relative inaccuracy in measurement of electron temperature and electron number density with used probes was ranging from 5% to 12% and 4% to 17%, respectively. The overall trend confirmed the highest values of electron temperature with an asymmetric double probe followed by a symmetric double probe and triple probe. The electron number density measured with a symmetric double probe was somewhat lower than those of the symmetric triple probe. The electron energy probability function was also investigated in these studies by using asymmetric probe; it was noticed that the high energy tail of electron energy probability function shrinks to the lower energies with the increase in filling gas pressure. It confirms the non-Maxwellian nature of the electron energy probability function and the presence of two groups of the energetic electrons at low pressures. The final outcomes of this experiment confirmed that the results obtained from electron temperature and electron number density by these three methods were very consistent, where a very good agreement was found among all measurements. It strengthens the concept of using these locally developed multitip probes for characterization of RF discharge plasma.
